# Microbiological Analysis Following Periodontal Treatment in Individuals with Bronchiectasis

**DOI:** 10.3390/microorganisms14051047

**Published:** 2026-05-06

**Authors:** Andreia La Selva, Ellen Sayuri Ando-Suguimoto, Ana Paula Mariano Santos Ginez, Tania Oppido Schalch, Renata Matalon Negreiros, Dione Kawamoto, Giuliana Giovinazzo Anselmo Ramos, Priscila Larcher Longo, Rodrigo Athanazio, Marcia Pinto Alves Mayer, Anna Carolina Ratto Tempestini Horliana

**Affiliations:** 1 Postgraduate Program in Biophotonics-Medicine, Universidade Nove de Julho (UNINOVE), São Paulo 01504-001, Brazil; dralaselva@gmail.com (A.L.S.); esa2406@gmail.com (E.S.A.-S.); anapmariano15@gmail.com (A.P.M.S.G.); taniaschalch@gmail.com (T.O.S.); renata.matalon@gmail.com (R.M.N.); dragiuliana20@gmail.com (G.G.A.R.); pllongo@gmail.com (P.L.L.); 2 Department of Microbiology, Institute of Biomedical Sciences, University of São Paulo, São Paulo 05508-000, Brazil; 77didi@gmail.com (D.K.); mpamayer@icb.usp.br (M.P.A.M.); 3 Postgraduate Program in Aging Science, São Judas Tadeu University (USJT), São Paulo 05503-001, Brazil; 4 Pulmonary Department, Heart Institute (InCor), School of Medicine, University of São Paulo, São Paulo 04023-062, Brazil; rathanazio@yahoo.com.br

**Keywords:** microbiological analysis, bronchiectasis, periodontitis, quality of life

## Abstract

Periodontal status has been associated with infection in lung diseases such as chronic obstructive lung disease (COPD). However, evidence regarding its association with bronchiectasis remains limited, despite the shared clinical and pathophysiological characteristics between the two conditions. Therefore, the aim of the present study was to investigate whether periodontal treatment affects not only the microbiota of saliva but also that of sputum and nasal secretions in individuals with bronchiectasis. This single-center, parallel-group randomized controlled clinical trial included forty-nine individuals with bronchiectasis, who were randomly allocated using a predefined randomization sequence with allocation concealment to a conventional group (*n* = 26) submitted to mechanical periodontal treatment plus oral hygiene and a control group (*n* = 23) submitted to oral hygiene alone. Due to the nature of the intervention, participants and operators were not blinded. At the end of the study, all participants received periodontal treatment. The primary outcome was the quantitative assessment of *Pseudomonas aeruginosa* (*P. aeruginosa*), *Staphylococcus aureus* (*S. aureus*), and *Porphyromonas gingivalis* (*P. gingivalis*) in sputum. Secondary outcomes included the quantification of these microorganisms in saliva and nasal secretions, as well as clinical periodontal parameters and quality-of-life assessment. All variables were evaluated at baseline and three months after treatment. Results: Periodontal treatment improved gingival and plaque indices in the conventional group compared with the control group. However, no significant differences were observed in sputum samples for any of the microorganisms analyzed, suggesting no measurable effect on bacterial levels in the lower airways within the study period. At the end of the experimental period, levels of *P. aeruginosa* and *P. gingivalis* decreased in nasal secretions, and levels of *P. aeruginosa* decreased in saliva in the conventional group but not the control group. No significant differences were found in *S. aureus* levels between groups or overtime. Also, no significant differences in total OHIP-14 scores were observed between groups. In conclusion, periodontal treatment was associated with reductions in *P. aeruginosa* in nasal secretions and saliva, and *P. gingivalis* in nasal secretions, in individuals with bronchiectasis and periodontitis. Periodontal treatment improved gingival and plaque indices in the conventional group compared with the control group. However, no significant differences were observed in sputum samples for any of the microorganisms analyzed, suggesting no measurable effect on bacterial levels in the lower airways within the study period. At the end of the experimental period, levels of *P. aeruginosa* and *P. gingivalis* decreased in nasal secretions, and levels of *P. aeruginosa* decreased in saliva in the conventional group but not the control group. No significant differences were found in *S. aureus* levels between groups or overtime. Also, no significant differences in total OHIP-14 scores were observed between groups.

## 1. Introduction

Periodontal disease (PD) is an infectious condition that affects the supporting tissues of the teeth [1]. PD has been associated with several systemic diseases, such as diabetes [2], cardiovascular diseases [3], rheumatoid arthritis [4], obesity [5], and metabolic syndrome [6]. This condition may also be a modifying factor in systemic health, and its clinical consequences may affect quality of life. The Oral Health Impact Profile (OHIP) can be used to assess the extent to which oral conditions interfere with quality of life and to better understand their impact [7]. Recent evidence [8] suggests that chronic periodontitis may also negatively impact quality of life and disease severity in individuals with bronchiectasis, demonstrating the importance of investigating oral health-related quality of life in this population.

Although associations between PD and lung diseases, such as pneumonia, asthma, and chronic obstructive pulmonary disease (COPD), have been suggested, causal relationships remain unclear [9,10,11]. A proposed pathogenic mechanism is that the aspiration of oral bacteria, including periodontopathogens such as *Porphyromonas gingivalis* (*P. gingivalis*) found in tracheal aspirates [11,12], may contribute to pulmonary infection, whereas mechanical control of the dental biofilm leads to improvements in clinical periodontal variables and can exert a positive influence by reducing the occurrence of pulmonary exacerbations in COPD [9,13,14,15].

Bronchiectasis is a chronic suppurative lung disease characterized by permanent and irreversible bronchial dilation. This condition causes significant morbidity and interferes with quality of life [16]. Due to similarities in clinical manifestations, such as cough and sputum production, bronchiectasis is often misdiagnosed as COPD [17]. The most common microorganisms in the lower airways of adults with bronchiectasis are *Pseudomonas aeruginosa* (*P. aeruginosa*) and *Staphylococcus aureus* (*S. aureus*) [18,19,20,21], both of which are often found in dental biofilm [14]. Colonization of the lower airways by *P. aeruginosa* is associated with a greater number of exacerbations and prolonged hospitalization [21]. During exacerbations, a combination of intravenous antibiotics constitutes the treatment of choice [22]. Macrolides reduce the frequency of exacerbations by inhibiting increases in sputum volume [23], although their mechanism of action involves immunomodulatory and antimicrobial effects [13]. Therefore, improving oral hygiene and reducing bacterial colonization of the oropharynx may help prevent pulmonary exacerbations [13].

Given this interaction between oral and respiratory environments, the analysis of saliva, nasal secretions, and sputum may provide complementary information on microbial distribution across interconnected anatomical sites.

The goal of oral hygiene counseling is to control dental biofilm, while professional mechanical treatment (scaling and root planing) is considered the gold-standard treatment for PD [1]. The present study was designed not to provide a comprehensive periodontal microbiological profile, but to investigate whether periodontal treatment could influence selected microorganisms across oral and respiratory-related secretions. Thus, a targeted, hypothesis-driven approach was adopted, focusing on specific microorganisms selected for their established relevance to both periodontal disease and bronchiectasis. Accordingly, specific microbiological targets were defined a priori, including *P. gingivalis* as a key periodontopathogen [24], together with *P. aeruginosa* and *S. aureus*, which are highly relevant in bronchiectasis [25,26], as well as their presence in oral and respiratory environments [27,28]. This rationale is supported by the concept of the oral–lung axis, in which oral dysbiosis may contribute to respiratory microbial colonization through microaspiration and microbial exchange between anatomical sites, as well as through shared inflammatory pathways between the oral cavity and lower airways [11,29]. Accordingly, the study focuses on the oral–respiratory interaction rather than on a classical periodontal microbiological profile alone. We hypothesized that periodontal mechanical treatment could reduce the levels of selected microorganisms across oral and respiratory-related secretions in individuals with bronchiectasis.

Therefore, the present study aimed to evaluate whether periodontal mechanical treatment may affect the microbiota of participants with bronchiectasis by analyzing sputum, nasal secretions, and saliva samples at baseline and at three months post-treatment.

## 2. Materials and Methods

The present prospective, randomized, controlled, parallel clinical trial was registered with clinicaltrials.gov (number NCT02514226) and received approval from the Human Research Ethics Committee of Universidade Nove de Julho (UNINOVE # 1057901), with the co-participation of the Pulmonary Department of the Heart Institute (InCor), School of Medicine, University of São Paulo, São Paulo, Brazil.

After receiving verbal and written clarifications, all volunteers signed an informed consent form before being included in the study. All participants had a diagnosis of bronchiectasis based on clinical history and chest computed tomography findings [16], were clinically stable, and were undergoing treatment at the outpatient clinic of the Discipline of Pulmonology of the Heart Institute (InCor), School of Medicine, University of São Paulo. The sample consisted of participants with bronchiectasis who were also diagnosed with periodontal disease [1]. The patients were referred for periodontal treatment at the UNINOVE Dental Clinic, São Paulo, Brazil, from November 2015 to November 2018.

The primary outcome was the level of *P. aeruginosa*, *S. aureus*, and *P. gingivalis* in the sputum, saliva, and nasal secretions of individuals with bronchiectasis. The secondary outcomes were clinical periodontal variables and quality of life, assessed using the Oral Health Impact Profile-14 (OHIP-14) questionnaire. All these variables were collected at baseline and after three months. Demographic data were also collected (age, sex, marital status, occupation, schooling, living conditions, and income).

The sample size was calculated a priori using G*Power software [29] (version 3.1.9.2; based on an F test for repeated-measures ANOVA with within–between interaction, considering two groups and four repeated measurements. The calculation was based on expected differences in microbiological outcomes, particularly sputum-related parameters. Previous studies have demonstrated an association between oral and respiratory pathogens in patients with chronic respiratory diseases, supporting the biological plausibility and clinical relevance of these outcomes. For instance, [15] reported similarities between subgingival and respiratory microbiota, while other studies have shown associations between airway bacterial load and inflammatory markers in COPD. However, as these studies were observational in nature and employed heterogeneous statistical approaches, they did not provide effect size estimates directly applicable to a repeated-measures design with group comparison. Therefore, an effect size of f = 0.40 was assumed, corresponding to a moderate effect according to Cohen’s conventions. The calculation assumed a significance level of α = 0.05 and a statistical power of 80% (β = 0.20), resulting in a minimum required sample size of 46 participants distributed between the two groups.

No formal adjustment for potential dropouts was made; however, the final sample exceeded the minimum required size. Due to the study design, which included a single follow-up assessment, a full intention-to-treat approach could not be adequately implemented for participants with missing follow-up data. The intraclass correlation coefficient (ICC) was calculated to determine intra-examiner agreement for clinical periodontal variables (ICC = 0.80). Standardization was also conducted on administering the OHIP-14 questionnaire and collecting secretions.

The inclusion criteria were a diagnosis of bronchiectasis at the Discipline of Pulmonology, Heart Institute (InCor), School of Medicine, University of São Paulo, São Paulo, Brazil, age > 28 years, either sex, having >10 teeth, and clinical probing depth (PD) ≥ 3 mm [30]. These criteria were defined to ensure the inclusion of individuals with periodontal involvement while allowing for the clinical variability typically observed in this population.

The exclusion criteria were the use of medications that modify periodontal status (phenytoin or cyclosporine), use of antiseptics in the previous 3 months, uncontrolled systemic diseases, prophylactic antibiotic therapy, use of anti-inflammatories in the previous month, periodontal treatment in the previous 6 months, smokers, and pregnant or lactating women. Patients with bronchiectasis secondary to cystic fibrosis, asthma, and chronic obstructive pulmonary disease (COPD) were excluded to obtain a more homogeneous study population and to minimize potential confounding effects associated with these conditions, which present distinct pathophysiological mechanisms, clinical courses, and management strategies [31,32]. All patients with bronchiectasis are routinely screened for etiologies such as cystic fibrosis, variable common immunodeficiency, allergic bronchopulmonary aspergillosis, and alpha-1 antitrypsin deficiency. Thus, all patients underwent a sweat test, and cystic fibrosis was excluded if chloride levels were below 30 mmol/L. If values were between 30 and 60, a genetic test (CFTR sequencing) was performed to exclude the disease. COPD was ruled out, since we did not include patients with a smoking history of 10 pack-years or more. We excluded patients whose bronchiectasis was attributed to asthma as the etiology, based on a long history of asthma and reversibility on spirometry.

A researcher not directly involved in the study generated the random allocation sequence in Microsoft Excel using block randomization with 10 fixed blocks of six participants, ensuring balanced group allocation throughout the recruitment period. Allocation concealment was ensured using sequentially numbered, sealed, opaque envelopes containing group assignments in the order defined by the randomization sequence. The envelopes were prepared in advance by an independent researcher (ACRTH), sealed, and stored in numerical order in a secure location, with access restricted to personnel responsible for allocation procedures. Following baseline assessment, all participants were evaluated by the study investigator using standardized procedures. The researcher responsible for the intervention retrieved and opened the next envelope in sequence to determine group allocation, strictly maintaining the numerical order of the envelopes. The intervention was then performed according to the assigned group, and only this researcher had access to the allocation information. Outcome assessments were conducted by an examiner (AL) before and after the intervention. Due to the nature of the interventions and inherent differences in treatment schedules between the control and conventional groups, blinding of participants and care providers was not feasible. Additionally, the examiner was not blinded to the group allocation because clinical periodontal conditions were readily distinguishable. Clinical periodontal assessments were performed by a calibrated examiner using standardized measurement criteria. The participants in the conventional group were scheduled for immediate treatment, and those in the control group were scheduled for treatment at three-month intervals. Despite this limitation, the primary outcomes were objective measures, thereby minimizing the risk of bias. These outcomes were based on quantifiable, reproducible criteria less influenced by the subjective perceptions of the participants and care providers.

The clinical trial was structured into two parallel groups: conventional (*n* = 26), which received scaling and root planing and oral hygiene counseling, and control (*n* = 23), which received oral hygiene counseling alone. Oral hygiene counseling included encouraging daily toothbrushing and the use of dental floss. The participants in the conventional group received conventional periodontal treatment with universal curettes (Hu-Friedy^®^) and ultrasound. Scaling and root planing (SRP) was performed in a single session by a single experienced researcher.

At baseline and three months after periodontal treatment, the participants in both groups answered a questionnaire for the assessment of the impact of oral health status on quality of life (OHIP-14 questionnaire), the biological samples were collected, and a clinical periodontal assessment was performed for the determination of probing depth (PD), clinical attachment level (CAL), bleeding on probing (BP), gingival index (GI), and plaque index (PI).

The OHIP-14 questionnaire was used to measure the impact of perceived oral problems on oral health-related quality of life [33,34]. The patient answered 14 questions by assigning responses of 0 (never), 1 (almost never), 2 (sometimes), 3 (most of the time), or 4 (always). The results were analyzed using the additive method, in which the points are summed (range: 0–56), with higher scores indicating a greater impact on quality of life.

The clinical periodontal assessment was performed by a calibrated examiner with a periodontal probe at six sites per tooth, excluding third molars [35]. Probing depth was measured in millimeters from the base of the periodontal pocket to the free gingival margin. Clinical attachment level was measured in millimeters from the cementoenamel junction to the base of the periodontal pocket. The gingival index, visible plaque index, and bleeding on probing were determined dichotomously [35].

Collection and processing of sputum, saliva, and nasal secretion. To collect sputum, the participants were instructed to rinse their mouths with water, remove the prosthesis, take several deep breaths, and cough deeply, followed by expectoration (5 mL) into a 50 mL centrifuge tube without touching the lips to the tube. Non-stimulated saliva (5 mL) was also collected in 50 mL tubes. Nasal secretion samples were collected using a sterile swab and stored in 500 μL of Tris-EDTA (TE). All samples were identified, packed on ice, and stored at −80 °C until analysis.

For sputum processing, 1 mL was withdrawn from the collection tube and transferred to a new tube. Next, 5 mL of 0.85% saline solution was added, followed by stirring to separate the sputum from the adherent saliva. The tube was centrifuged at 4200× *g* and 4 °C for 10 min (Eppendorf^®^ 5804 centrifuge). The saline solution was removed. This washing step was performed to reduce contamination from saliva and upper airway secretions. An equal volume of Remel^TM^ Sputasol solution (dithiothreitol 0.54%) was added to the washed sputum. The mixture was incubated at 37 °C with periodic stirring at 1 g (60 rpm) for 15 min and vortexed every 5 min. At the end of the process, the solution was stored at −80 °C. Saliva was centrifuged at 2057× *g* and 4 °C for 10 min, and the supernatant was discarded. The precipitate was resuspended in 500 μL of TE and stored at −80 °C.

DNA extraction was performed with Meta-G-Name™ DNA Isolation-MGN0910 (Epicenter Technologies Corp., Chicago, IL, USA). DNA samples were stored in a freezer at −20 °C and quantified using a spectrophotometer (Nanodrop ND1000—Thermo Fisher Scientific Inc., Waltham, MA, USA).

The quantification of *P. gingivalis*, *P. aeruginosa*, and *S. aureus* was performed by real-time PCR (qPCR) using species-specific primer pairs (Table 1). The reaction consisted of 2 μL of DNA, 25 pMol of each primer, and 10 μL Sybr Green (Quantimix Easy SYG Kit, Applied Biosystems, Waltham, MA, USA). Quantification was performed in the Step One Plus Thermal Cycler (Applied Biosystems). The cycles are described in Table 1.

Standard curves were generated using serial dilutions of DNA templates with known concentrations, and the corresponding copy numbers were calculated based on molecular weight, considering the length (in base pairs) of the amplified fragment and Avogadro’s constant. Serial dilutions ranging from 10^1^ to 10^8^ copies were used to construct the standard curves. All qPCR reactions were performed in triplicate to ensure reproducibility and reliability of the measurements.

The standard curve consisted of serially diluted 16S rRNA templates ranging from 10 to 10^8^ copies of *P. gingivalis* ATCC 33277, *P. aeruginosa*, and *S. aureus*. Reaction efficiency was considered 100 ± 10%.

The results were expressed in quantity of bacteria for *P. gingivalis*, *P. aeruginosa*, and *S. aureus* (16S rRNA quantity values divided by the number of 16S rRNA copies presented in the gene of each bacterium: four copies for *P. gingivalis* and *P. aeruginosa* and six copies for *S. aureus* (https://rrndb.umms.med.umich.edu/ accessed on 12 July 2018).

Bacterial quantification was based on an absolute quantification approach, allowing conversion of CT values into copy numbers using the standard curves. Bacterial levels were expressed as absolute quantitative values derived from qPCR analysis. Logarithmic transformation was applied only for graphical representation, to improve visualization of data distribution, while all statistical analyses were performed using the original (non-transformed) data. No internal amplification control or extraction control was included in the qPCR protocol, as the objective of the study was the absolute quantification of bacterial load rather than gene expression analysis. However, this should be considered a methodological limitation.

The Shapiro–Wilk test was used to assess the normality of the data. Group comparisons were performed for all variables using the Mann–Whitney test. The data were expressed as median (first quartile [Q1]; third quartile [Q3]), highlighting central tendency and variability. A logarithmic transformation was applied only for the graphical representation of microbiological results, while statistical analyses were performed on the original (non-transformed) data. For age, which presented a normal distribution, comparisons between groups were made. For OHIP, the Wilcoxon test was also used. A *p*-value < 0.05 was initially considered statistically significant. To reduce the risk of type I error in the primary microbiological analyses, *p*-values were further adjusted using the Benjamini–Hochberg false discovery rate procedure (clinical and microbiological analyses). Adjusted *p*-values were considered significant at q < 0.05. Data analysis was performed using IBM SPSS Statistics (version 22).

## 3. Results

**Figure 1:**
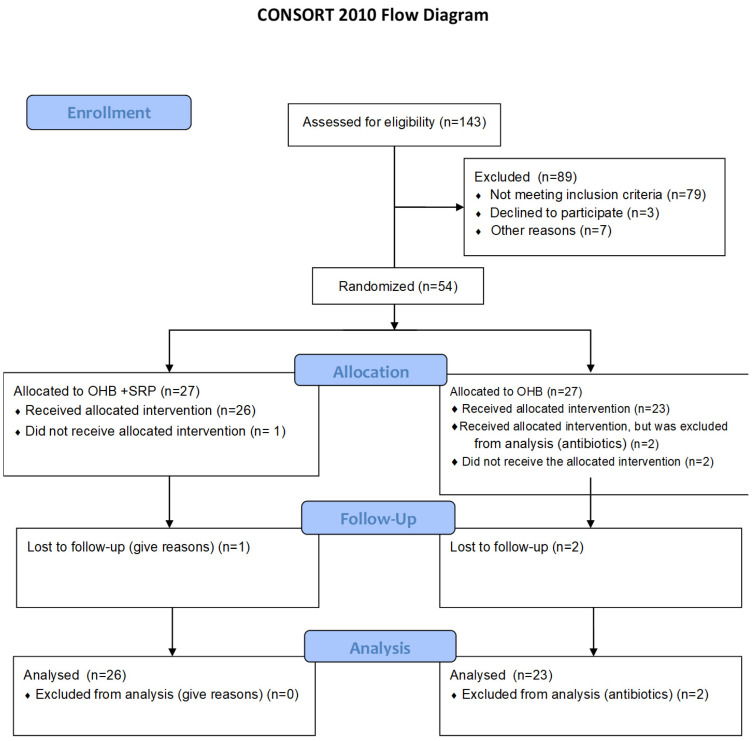
CONSORT flowchart of study.

A total of 143 patients with bronchiectasis were screened. Eighty-nine individuals were excluded from the study due to the following: absence of more than 10 teeth with a probing depth greater than 3 mm, use of complete dentures, use of antibiotics other than macrolides, use of anti-inflammatory drugs in the previous month, periodontal treatment in the previous six months, or use of mouthrinses. Thus, only 54 individuals were randomized. One patient in the treatment group and two in the control group did not receive the allocated interventions due to long distances, lack of oxygen cylinder autonomy, limited mobility, etc. Two patients received the allocated intervention but were excluded from the analysis for taking antibiotics other than macrolides (*n* = 2) (Figure 1). Therefore, the final analysis was performed using a per-protocol approach, including only participants who completed the study as prescribed.

### 3.1. Demographic Data

Mean age was 51.69 ± 11.56 years in the overall sample (50.5 ± 11.56 years in the control group and 52.8 ± 11.35 years in the conventional group). No significant difference in mean age was found between groups (*p* = 0.488). No statistically significant differences were observed between the groups for the variables analyzed (Table 2).

### 3.2. Microbiological Analysis

Table 3 shows the median (Q1; Q3) concentrations of *P. gingivalis*, *P. aeruginosa*, and *S. aureus* in biological secretions (nasal, saliva, and sputum) investigated at baseline and after three months in the control and conventional groups. The levels of *Porphyromonas gingivalis* (*p* = 0.001) and *Pseudomonas aeruginosa* (*p* = 0.0082) in nasal secretions decreased in the conventional group between baseline and three months after treatment. Although statistically significant, the observed reduction corresponded to a modest absolute change.

**Figure 2:**
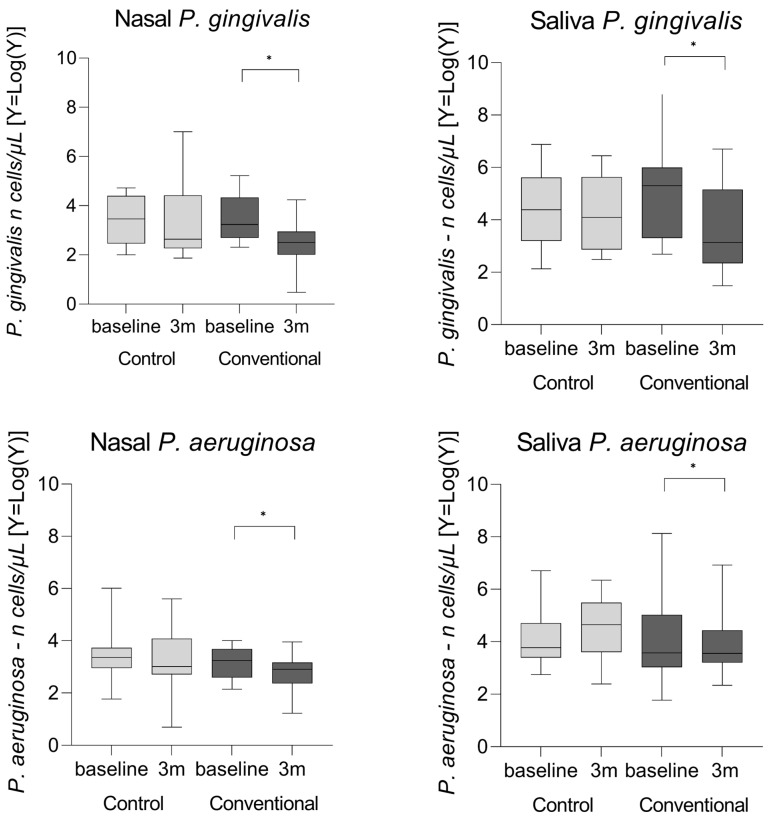
Representation of *P. gingivalis* and *P. aeruginosa* copies in nasal and salivary samples. Graphs show logarithmic values (Y = log(Y)) of bacterial copies in two experimental groups (control and conventional) assessed at baseline and after three months (3 m). Log transformation was applied for visualization purposes only, and statistical analyses were performed using the original (non-transformed) data. Asterisks (*) indicate statistically significant differences after false discovery rate (FDR) correction. Values are expressed as median and interquartile range. Data were compared between groups using the Mann–Whitney test. After FDR adjustment, all previously significant results remained significant, except for salivary *P. gingivalis*.

In saliva, a significant difference was found between the conventional and control groups for *P. aeruginosa* (*p* = 0.0067) when comparing baseline to the three months after treatment (Table 3 and Figure 2). Although statistically significant, the observed difference corresponded to a modest absolute change. After false discovery rate adjustment of the primary microbiological comparisons, the reductions in nasal *P. gingivalis* and in nasal and salivary *P. aeruginosa* remained statistically significant, whereas the reduction in salivary *P. gingivalis* did not.

### 3.3. Clinical Periodontal Variables

**Figure 3:**
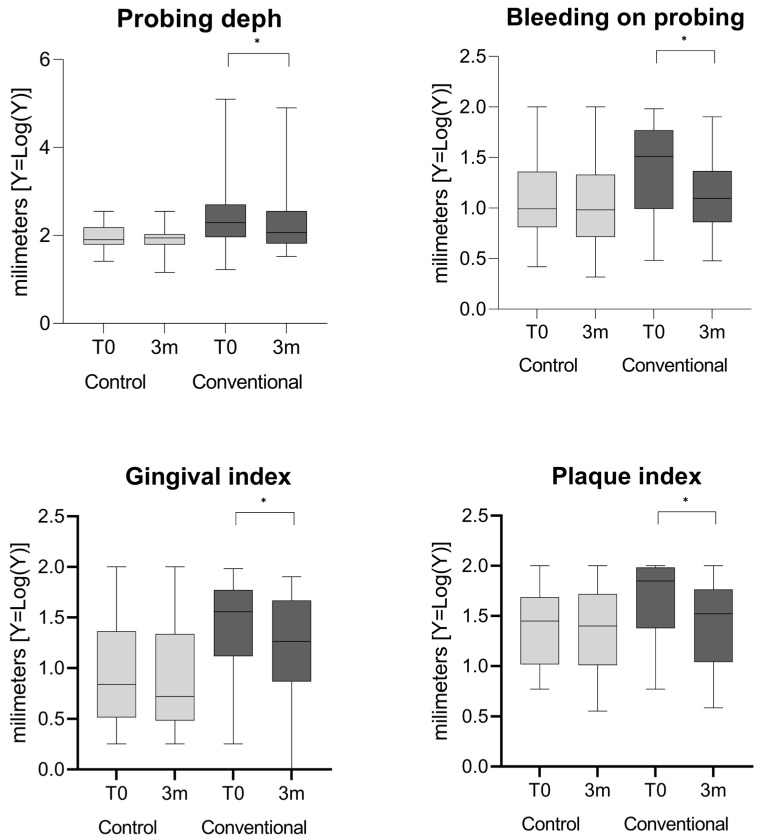
Clinical periodontal variables, including pocket depth (PD), clinical attachment level (CAL), gingival index, and plaque index, in control and conventional groups at baseline (T0) and after three months (3 m). Data are presented as median and interquartile range (Q1–Q3). Graphs display logarithmically transformed values for visualization purposes only, while statistical analyses were performed using the original (non-transformed) data. Statistical significance was evaluated after false discovery rate (FDR) correction, and significant differences are marked with asterisks (*).

Table 4 shows differences between PD, GI, and PI after periodontal treatment. Although statistically significant improvements were observed in the conventional group, the reductions were modest in absolute terms. Periodontal treatment resulted in improvements in probing depth, bleeding on probing, gingival index, and plaque index in the conventional group compared to the control group. However, after false discovery rate (FDR) adjustment, only the gingival index and plaque index remained statistically significant. No reduction was found in any of these variables in the control group. The variables were compared between baseline and three months after treatment (Table 4 and Figure 3).

The distribution across severity stages followed the 2017 World Periodontitis Classification. Eight patients in Stage 1 exhibited initial periodontitis, characterized by mild clinical attachment loss and localized inflammation. Six patients in Stage 2 were diagnosed with moderate periodontitis, marked by more significant attachment loss and potential bone involvement. Two patients were identified in Stage 3, both showing signs of severe periodontitis, including deep periodontal pockets and dental mobility. Stage 4 (most advanced stage) comprised 11 patients, distributed among categories 4A, 4B, and 4C, characterized by extensive bone loss and high risk of tooth loss.

In addition to cases of periodontitis, six patients presented biofilm-induced gingivitis in a reduced periodontium, with four cases classified as generalized and two as localized. Notably, 16 patients were classified as having clinical gingival health in a reduced periodontium, indicating periodontal stability despite a previous history of disease. These findings highlight the variability in the progression of periodontitis and the importance of personalized approaches for the prevention and treatment of periodontal disease.

### 3.4. OHIP-14 Questionnaire

In the control group, total OHIP-14 scores increased slightly from baseline to 3 months, but the change was not statistically significant (*p* = 0.94). In the conventional treatment group, a small reduction in OHIP-14 scores was observed over time, but this change was also not statistically significant (*p* = 0.76).

No significant differences were observed between groups at baseline (*p* = 0.50) or after 3 months (*p* = 0.59). Additionally, the change in OHIP-14 scores over time did not differ significantly between groups (*p* = 0.77). Although small variations were observed, the magnitude of change was modest, which may limit clinical relevance and should be interpreted with caution.

## 4. Discussion

As periodontal diseases have been associated with the development and exacerbation of pulmonary diseases, it is important to investigate the relationship between periodontitis and bronchiectasis [40]. This study investigated the influence of conventional periodontal treatment on bacterial levels in saliva, sputum, and nasal secretions, as well as on clinical periodontal variables and patient quality of life. Briefly, we observed decreases in the levels of *P. aeruginosa* and *P. gingivalis* in nasal secretions following periodontal treatment as well as decreases in the levels of *P. gingivalis* and *P. aeruginosa* in the saliva of these participants. As expected, some clinical periodontal variables improved three months after periodontal treatment, with reductions in the gingival index and the plaque index. Despite these clinical improvements, no differences were found in total OHIP-14 scores between groups three months after therapy.

Bronchiectasis is a severe pulmonary disease with frequent exacerbations, hospitalizations, and recurring infections [41,42]. Patients with bronchiectasis may suffer up to 12 exacerbations per year, with an average of 1 to 3. The occurrence and frequency of exacerbations have been related to multiple clinical and socioeconomic factors [18]. Furthermore, the phenotype of patients more prone to exacerbations includes those with chronic *P. aeruginosa* infection [43]. More than 143 patients were screened in the present study. The exclusion criteria were antibiotic use, residence in different cities, the absence of periodontitis, the use of complete dentures, and fewer than ten remaining teeth. Bronchiectasis may affect young people and adults, with no preference for gender or ethnic group [44]. The mean age of participants was 51.7 ± 11.6 years, and sociodemographic characteristics were consistent with previous reports in populations receiving care within the Brazilian Unified Health System [45].

Some studies have reported correlations between pulmonary diseases and periodontitis [13,46]. The experimental hypothesis of this study was that periodontal treatment would reduce levels of *P. aeruginosa* and *S. aureus* in certain secretions. Previous studies have detected *P. aeruginosa* in saliva [27,47], nasal secretions [48], and sputum [49,50], as well as *S. aureus* in saliva [51], nasal secretions [26], and sputum [52] among patients with bronchiectasis. The presence of these bacteria increases morbidity and mortality [53,54]. In our study, periodontal treatment decreased the quantity of *P. aeruginosa* in nasal secretions and saliva after three months. However, no significant changes were observed in sputum samples, which represent the lower airway compartment and are more directly related to disease activity in bronchiectasis. This finding is particularly relevant, as it indicates that periodontal treatment did not result in measurable changes in bacterial colonization of the lower airways within the study period for the three targeted species analyzed (*P. aeruginosa*, *S. aureus*, and *P. gingivalis*), highlighting the limitations of a single-pathogen approach. Importantly, these findings should be interpreted within the context of current concepts of microbial dysbiosis, in which respiratory diseases such as bronchiectasis are characterized by complex, polymicrobial communities and dynamic host–microbiome interactions rather than the presence or absence of individual pathogens [20] Recent advances in the understanding of the oral–lung axis further support the notion that microbial exchange between anatomical compartments involves ecological shifts in microbial communities, which may not be adequately captured by targeted analyses of selected species [12,20]. Therefore, these results indicate that the observed microbiological changes may be restricted to upper airway-related compartments, and that broader microbiome-based approaches—capable of capturing complex microbial communities and ecological shifts—may be necessary to detect potential changes in the lower airways. In addition, the relatively short follow-up period may have limited the detection of delayed or cumulative effects of periodontal intervention on respiratory microbiota. Furthermore, the present study was not designed to determine the directionality of bacterial transmission between anatomical sites. Therefore, it is not possible to establish whether *P. aeruginosa* detected in saliva and nasal secretions constitutes a reservoir for lung colonization or whether its presence in the oral cavity results from the expectoration of sputum via the oropharynx. It was not possible to determine whether patients with bacteria detected in saliva also presented the same microorganisms in sputum samples.

Although periodontal pockets have been described as potential niches for pathogenic bacteria [11], more recent evidence supports the role of the oral microbiome in shaping lung microbial communities and contributing to airway inflammation [12]. In this context, saliva may act as a vehicle for lung infection [48]. *P. aeruginosa* is strongly associated with pulmonary exacerbations in bronchiectasis and exhibits multiple virulence mechanisms, including biofilm formation and antibiotic resistance [55,56,57]. It can colonize upper airway sites such as the nasal cavity and sinuses prior to lung involvement [54,58].

Recent advances in the study of the airway microbiome in bronchiectasis have shifted the field from culture-based approaches toward sequencing-based methodologies, including 16S rRNA gene sequencing, shotgun metagenomics, and long-read sequencing technologies [20,59,60]. These approaches have demonstrated that bronchiectasis is characterized by heterogeneous and dynamic microbial communities rather than the presence of isolated pathogens. In this context, reduced microbial diversity and dominance by specific taxa have been consistently associated with worse clinical outcomes, including increased disease severity, exacerbation frequency, and mortality [59].

Among these taxa, *P. aeruginosa* has emerged as the most consistent and clinically relevant pathogen across microbiome-based studies, being strongly associated with reduced diversity and more severe disease phenotypes [59,60]. These findings reinforce the relevance of including *P. aeruginosa* as a primary target in the present study, as it represents not only a key respiratory pathogen but also an indicator of microbiome dysbiosis and disease progression. In contrast, *Staphylococcus aureus*, although frequently detected in culture-based studies and large clinical registries, appears to play a less central role in structuring airway microbial communities in sequencing-based analyses, where it is less consistently associated with dominant microbiome profiles or disease-driving clusters [61]. This distinction highlights the importance of interpreting targeted microbiological findings within the broader ecological context of the airway microbiome.

With regard to *P. gingivalis*, direct evidence from bronchiectasis airway microbiome studies remains limited. However, emerging data from microbiome and gut–lung axis studies indicate that oral-associated taxa, including *Porphyromonas* species, may be present in the lower airways and associated with host inflammatory responses [62]. In addition, studies in related respiratory conditions have demonstrated the presence of *P. gingivalis* in lung microbiota, supporting the concept of oral–lung microbial exchange and reinforcing its biological plausibility in the present study context. Importantly, recent metagenomic studies have shown that bronchiectasis microbiomes can be stratified into distinct ecological profiles, including *Pseudomonas*-dominated communities, *Haemophilus influenzae*-dominated profiles, and more diverse polymicrobial states, as well as patterns characterized by depletion of commensal taxa [60]. These findings reinforce that disease progression is associated not only with the presence of specific pathogens but also with broader disruptions in microbial community structure.

Taken together, these data indicate that, although the present study adopted a targeted approach focusing on three clinically relevant species, such an approach captures only a limited dimension of the airway microbiome. Therefore, the absence of significant changes in sputum samples may reflect the inability of targeted analyses to detect community-level ecological shifts. Future studies employing comprehensive sequencing-based approaches will be essential to further elucidate the impact of periodontal interventions on the respiratory microbiome.

Continuous antibiotic therapy is the treatment of choice for bronchiectasis [53]. Strategies to improve host defense and limit excessive inflammation may be important for improving the prognosis of pulmonary infections caused by *P. aeruginosa* [54]. Pulmonary infection caused by *Staphylococcus aureus* is challenging to treat due to its high resistance to several antibiotics [63]. In our study, periodontal treatment did not decrease *S. aureus* concentrations in any of the secretions. Both microorganisms have been found in oral biofilm [14].

The periodontopathogen *P. gingivalis*, considered a key contributor to periodontitis [64,65], has been identified in high quantities in tracheal aspirates [14]. Based on this, we hypothesized that periodontal treatment, supported by improved oral hygiene, could reduce salivary levels of *P. gingivalis*, a potential source for pulmonary aspiration. Although a reduction in salivary *P. gingivalis* was observed three months after periodontal treatment, this finding did not remain statistically significant after adjustment for multiple comparisons. A reduction was observed in the number of copies in the nasal secretion. It is important to note that *P. gingivalis* was detected alongside *P. aeruginosa* in tracheal aspirates from patients with acute exacerbations of COPD [14] and may increase the pathogenicity of *P. aeruginosa* in the lower airways [66]. Both bacteria invade respiratory epithelial cells, thereby reducing apoptotic events. This mechanism could provide bacteria with a safe intracellular niche and the opportunity to establish infection [66,67,68].

This study has some limitations, including the exclusion of hospitalized patients in the exacerbation phase. In addition, we should assume that all participants are under constant medical care and are taking azithromycin continuously, a macrolide with high evidence [69] of reducing the production of inflammatory cytokines, including IL-8, IL1β, and IL-17. This event decreases the concentration of Th17 cells in peripheral blood, which confers both immune stimulation in the lungs and periodontal protection [70]. It has been shown that periodontal treatment and oral hygiene can improve exacerbations in patients with COPD [51]. Indeed, the association between oral biofilm control and the severity of pulmonary diseases has been demonstrated [15]. However, the present study did not investigate clinical outcomes such as exacerbation frequency and, therefore, no conclusions can be drawn regarding the impact of periodontal treatment on the progression of bronchiectasis. Clinical trials are needed to confirm whether this premise applies to bronchiectasis. Additionally, the relatively small sample size may limit the statistical power to detect differences, particularly in sputum samples. Moreover, the use of a per-protocol analysis may introduce selection bias, as only participants who completed the study were included in the final analysis.

A limitation of the present study is that the microbiological analysis was restricted to *P. aeruginosa*, *S. aureus*, and *P.gingivalis*, and did not include a broader panel of respiratory and classical periodontopathogenic microorganisms commonly identified in bronchiectasis, such as *Haemophilus influenzae*, *Moraxella catarrhalis*, and *Streptococcus pneumoniae*. The selected targets were defined a priori based on their relevance to the study hypothesis, which focused on the oral–respiratory interface. In this context, the selected microorganisms were required not only to be involved in the etiology of lung infections but also to be commonly detected in the oral cavity. *P. aeruginosa* is one of the most prevalent pathogens in bronchiectasis and has been associated with increased disease severity and exacerbations [16]. Importantly, previous studies have demonstrated that this microorganism can also be detected in subgingival biofilm and in the oral cavity of patients with pulmonary diseases [25,71,72], supporting its potential role in the oral–respiratory microbial interplay. Similarly, *S. aureus*, although not considered a classical periodontopathogen, has been identified in subgingival sites and associated with periodontitis [28], as well as detected within oral epithelial cells [73], suggesting its ability to persist in oral niches and potentially contribute to cross-site colonization. *P. gingivalis* was included as a keystone [24] pathogen in periodontal disease and a representative organism of periodontal dysbiosis. In addition to its well-established role in periodontitis, experimental and clinical studies have shown that this species can be detected in respiratory samples and may contribute to inflammatory responses in the lung [74,75,76,77,78], reinforcing its relevance in the oral–respiratory axis. On the other hand, respiratory pathogens commonly identified in bronchiectasis, such as *Haemophilus influenzae*, *Moraxella catarrhalis*, and *Streptococcus pneumoniae*, are usually not detected in subgingival sites. Thus, the present study focused on these three microorganisms because they are relevant to the study hypothesis and amenable to targeted analysis. We acknowledge that the inclusion of a broader panel of periodontopathogenic species or a more comprehensive microbiome analysis could have expanded the periodontal interpretation of the findings. Therefore, future studies should consider expanded microbiological panels, including multiple periodontopathogenic and respiratory species, to further explore the relationship between periodontal disease and bronchiectasis.

The aim of periodontal treatment is to control infection, prevent bone loss, and reduce excessive inflammatory responses [79] through scaling and root planing. Combined with improved oral hygiene, this therapy can reduce inflammation and probing depth, thereby improving periodontal status [1]. As expected, the participants in the study exhibited improvements in some clinical variables (GI, and PI) after three months, demonstrating the efficacy of periodontal treatment. However, the magnitude of change in periodontal parameters was modest, which may limit their clinical relevance. This finding should be interpreted with caution, particularly considering the short follow-up period.

Recent studies have shown that periodontal disease is associated with reduced quality of life, particularly due to physical discomfort. In the present study, no significant differences in total OHIP-14 scores were observed between groups after three months. This finding suggests that, despite improvements in clinical periodontal parameters, the magnitude of change was insufficient to translate into perceptible improvements in patient-reported outcomes within the study period.

Previous studies have reported consistent reductions in OHIP-14 scores after periodontal therapy, often reaching single-digit values, especially in patients with higher baseline severity. In contrast, the relatively low baseline scores observed in the present study may have limited the potential for detectable improvement. This may be related to the clinical profile of the sample, including regular medical follow-up and the use of macrolide antibiotics, which may have reduced the baseline impact of oral conditions. Additionally, the OHIP-14 is a generic instrument and may lack sensitivity to detect subtle changes specifically related to periodontal treatment, particularly in populations with low baseline OHIP-14 scores.

Importantly, although the OHIP-14 is widely used and considered a standard instrument for assessing oral health-related quality of life, it is not specific to periodontitis and may not be sufficiently sensitive to capture changes directly related to periodontal conditions or their treatment. This limitation should be considered when interpreting the absence of significant changes in patient-reported outcomes. Additionally, when the results are considered across outcome domains, a lack of concordance is observed between microbiological findings, clinical periodontal improvements, and patient-reported outcomes. While reductions in *P. aeruginosa* and *P. gingivalis* were detected in upper airway-related compartments, and improvements were observed in clinical parameters such as gingival index and plaque index, these changes were not accompanied by significant improvements in OHIP-14 scores.

This pattern suggests that microbiological and clinical improvements—particularly when modest and compartment-specific—may not be sufficient to generate measurable benefits in patient-perceived quality of life within the study period. These findings reinforce the complexity of the relationship between local microbial changes, periodontal clinical outcomes, and systemic patient perception, highlighting that improvements in surrogate biological markers do not necessarily translate into clinically meaningful benefits from the patient’s perspective. Furthermore, contemporary clinical research emphasizes the importance of patient-reported outcomes as essential endpoints. However, the present results indicate that more specific and sensitive instruments, particularly those tailored to periodontal disease, may be required to adequately capture changes in patient perception following periodontal treatment.

A limitation of this study is the absence of a formal adjustment for potential dropouts in the sample size calculation. In addition, due to the study design, which included a single follow-up assessment, a full intention-to-treat approach could not be adequately implemented, as participants with missing follow-up data would contribute only baseline information. To mitigate this limitation, the final sample size exceeded the minimum required number estimated in the a priori calculation, reducing the potential impact of missing data on the study findings. Although the FDR correction was applied, the risk of type I error cannot be completely excluded, given the number of comparisons performed. Another limitation was that adherence to oral hygiene instructions was not formally monitored, which may have influenced the outcomes and represents a potential confounding factor when interpreting the effects of the intervention.

It is important to note that the inclusion and exclusion criteria, particularly the exclusion of patients with COPD and smokers, may limit the external validity of the findings. However, these criteria were intentionally adopted to reduce potential confounding factors and to ensure a more homogeneous study population. Additionally, the use of a probing depth ≥ 3 mm as an inclusion criterion may have introduced some degree of periodontal heterogeneity, although it reflects the clinical variability commonly observed in this population.

The main objective of treatment for bronchiectasis is to reduce the bacterial load and inflammation, which results in improvements in symptoms, exacerbations, and quality of life [46]. As periodontal treatment aims to control bacterial infection by preventing disease progression [79], the present findings suggest that its effects may be limited to upper airway-related compartments, without evidence of impact on lower airway microbiology or clinically relevant pulmonary outcomes. Therefore, its potential systemic implications should be interpreted with caution, as the present study did not demonstrate changes in sputum samples or assess pulmonary function or exacerbation outcomes. These results position the present study as a targeted, hypothesis-driven investigation of the oral–respiratory interface, contributing to the understanding of compartment-specific microbial interactions rather than demonstrating a direct clinical effect on bronchiectasis progression.

This study contributes to the current literature by providing evidence of compartment-specific microbiological changes following periodontal treatment in individuals with bronchiectasis, highlighting a potential oral–respiratory interaction that is not reflected in lower airway microbiology. To our knowledge, this is one of the first randomized clinical trials to evaluate the impact of periodontal treatment across multiple respiratory-related compartments in bronchiectasis, providing novel insights into compartment-specific microbial responses.

## 5. Conclusions

Conventional periodontal treatment was associated with reductions in *P. aeruginosa* levels in nasal secretions and saliva, and in *P. gingivalis* levels in nasal secretions. No significant changes were observed in sputum samples. These findings should be interpreted with caution, as the observed microbiological changes were restricted to upper airway-related compartments, with no significant effects in sputum samples and no evidence of impact on clinical outcomes.

## Figures and Tables

**Table 1:** Sequences and references of primers employed.

Bacterium	Primer Sequence (5′–3′)	Amplification Conditions	Reference
*P. gingivalis* ATCC33277	F: TGTAGATGACTGATGGTGAAAACC R: ACGTCATCCCCACCTTCCTC	Initial denaturation: 95 °C for 10 min 40 cycles: 95 °C for 15 s, 60 °C for 1 min Melt curve: 83 °C for 10 s	Amano et al., 1999 [36] Tran; Rudney 1996 [37]
*P. aeruginosa* ATCC 10145T	F: GGCGTGGGTGTGGAAGTC R: TGGTGGCGATCTTGAACTTCTT	Initial denaturation: 95 °C for 10 min 40 cycles: 95 °C for 15 s, 60 °C for 1 min Melt curve: 83 °C for 10 s	Lee, 2011 [38]
*S. aureus* ATCC 25923	F: TCGAAATTAAATGTTATCGTGTCTTC R: TCGCGACATTCATTATGCCCAAATTTTTAA	Initial denaturation: 95 °C for 2 min 40 cycles: 95 °C for 15 s, 60 °C for 1 min	Goto, 2007 [39]

**Table 2:** Baseline Characteristics of Study Participants.

Variables	Distribution	Control	Conventional	*p*
		*n* = 23	*n* = 26	
Age		50.47 (±11.55)	52.76 (±11.35)	0.717 ^a^
Race/skin color	White	18 (78%)	13 (50%)	0.098 ^b^
	Black	4 (17%)	12 (46%)	
	Others	1 (4%)	1 (4%)	
Sex	Female	10 (43%)	11 (42%)	1.000 ^b^
	Male	13 (57%)	15 (58%)	
Marital status	Married	11 (47%)	12 (46%)	0.835 ^b^
	Divorced/Widowed	3 (13%)	5 (19%)	
	Single	9 (39%)	9 (34%)	
Income (multiples of monthly min. wage)	1 to 2	12 (52%)	19 (73%)	0.280 ^b^
	3 to 4	9 (39%)	5 (19%)	
	Five or more	2 (8%)	2 (7%)	

Table summarizes baseline characteristics of study participants, including age, skin color, sex, marital status, income (using monthly minimum wage as reference, monthly minimum wage = ±220 US dollars), and schooling in control and conventional groups. Continuous variables (e.g., age) expressed as mean ± standard deviation; categorical variables expressed as absolute number and percentage. Statistical significance assessed using: ^a^—Student’s *t*-test for age; ^b^—Pearson’s chi-square test for categorical variables.

**Table 3:** Comparison of Concentrations of Bacterial Species in Biological Secretions: Baseline and 3-Month Assessment in Control and Conventional Groups.

Secretion	Bacterial Species	Control			Conventional		
		Baseline	3 Months	*p*	Baseline	3 Months	*p*
Nasal	*P. gingivalis*	574.0	402.0	0.0715	1408.0	217.0	0.001 *
	IQR	(240.0–12,581.0)	(140.0–1182.0)		(493.5–13,887.0)	(14.0–683.0)	
	*P. aeruginosa*	2062.0	1620.5	0.0704	1748.5	497.5	0.0082 *
	IQR	(919.0–5070.0)	(251.0–9918.25)		(428.25–4335.0)	(15.75–1153.75)	
	*S. aureus*	1929.5	2217.0	0.8831	729.0	1573.0	0.5686
	IQR	(475.75–9114.75)	(1376.5–2341.5)		(201.5–1968.0)	(1245.5–2173.0)	
Saliva	*P. gingivalis*	4750.5	5094.0	0.8572	80,016.0	1210.0	**0.0426 ^&^**
	IQR	(147.0–323,436.0)	(537.5–292,254.0)		(489.0–767,988.0)	(164.5–42,619.0)	
	*P. aeruginosa*	5050.0	44,671.0	0.1548	3414.5	831.5	0.0067 *
	IQR	(2313.5–23,980.5)	(18,170.0–296,910.25)		(983.75–57,562.5)	(0.0–8839.75)	
	*S. aureus*	9244.0	4511.0	0.3824	11,629.0	4616.0	0.1266
	IQR	(4200.5–104,973,256,515.25)	(1019.0–28,011.0)		(4937.0–79,660,143,821.5)	(2217.0–75,623.0)	
Sputum	*P. gingivalis*	274,051,768,320.0	9,957,195,520.0	0.4988	748.5	507.0	0.8499
	IQR	(274,051,768,320.0–274,051,768,320.0)	(4,978,597,760.0–14,935,793,280.0		(243.0–8269.0)	(0.0–137,005.0)	
	*P. aeruginosa*	19,314,003.0	156.0	0.0711	2527	7140	0.2392
	IQR	(1733.5–156,505,822.5)	(2.0–543.0)		(338.5–6,347,702.5)	(0.0–403,637.5)	
	*S. aureus*	48,151,304.0	2687.0	0.0655	1840.5	1523.0	0.9115
	IQR	(203,102.5–13,793,523,093.75)	(488.5–611,316,409.5)		(16.25–4,978,506,975.0)	(283.5–911,714,200.5)	

Results are expressed as median and interquartile range (Q1; Q3) of concentrations of *P. gingivalis*, *P. aeruginosa*, and *S. aureus* in biological secretions (nasal, saliva, and sputum) assessed at baseline and after three months. Comparisons were performed between control and conventional groups. *p*-values are unadjusted; statistical significance was determined after false discovery rate (FDR) correction. * Refers to significant *p* value and ^&^ refers to no significant *p* values. ^&^ After FDR adjustment, all previously significant results remained significant, except for salivary *P. gingivalis*.

**Table 4:** Periodontal Outcomes: Comparison Between Control and Conventional Groups at Baseline and 3-Month Follow-Up.

Periodontal Outcomes	Group	Wilcoxon Test		
		Baseline	3 Month	*p*
Probing depth (PD)	Control	2.03	2.02	0.5781
	IQR	(1.89–2.39)	(1.83–2.35)	
	Conventional	2.46	2.07	0.0452 ^&^
	IQR	(2.00–3.07)	(1.84–2.52)	
Clinical attachment level (CAL)	Control	2.42	2.44	0.999
	IQR	(2.10–3.30)	(2.04–3.30)	
	Conventional	2.69	2.07	0.5380
	IQR	(2.29–3.33)	(1.84–2.52)	
Bleeding on probing (BP)	Control	10.06	9.08	0.8750
	IQR	(5.47–25.37)	(3.55–16.09)	
	Conventional	25.80	11.09	0.0315 ^&^
	IQR	(8.01–53.86)	(7.07–22.06)	
Gingival index (GI)	Control	2.29	2.29	0.9999
	IQR	(0.00–14.72)	(0.00–10.92)	
	Conventional	19.58	2.17	0.0063 *
	IQR	(0.00–50.57)	(0.00–20.26)	
Plaque index (PI)	Control	15.28	20.41	0.4375
	IQR	(7.00–38.72)	(8.75–38.72)	
	Conventional	66.18	28.67	0.0021 *
	IQR	(21.10–95.27)	(9.87–55.77)	

The table presents periodontal outcomes—probing depth (PD), clinical attachment level (CAL), bleeding on probing (BP), gingival index (GI), and plaque index (PI)—in control and conventional groups at baseline and after three months. Data are expressed as median and interquartile range (Q1; Q3). BP, GI, and PI are expressed as percentages; PD and CAL are in millimeters. *p*-values were calculated using the Wilcoxon test. ^&^ *p*-values are unadjusted; statistical significance was evaluated after false discovery rate (FDR) correction. * Refers to significant *p* value and ^&^ refers to no significant *p* values. After FDR adjustment, only the gingival index (GI) and plaque index (PI) remained statistically significant.

## Data Availability

The raw data supporting the conclusions of this article will be made available by the authors on request.
